# The Importance of MRI in the Early Diagnosis of Acute Invasive Fungal Rhinosinusitis

**DOI:** 10.3390/diagnostics15030311

**Published:** 2025-01-28

**Authors:** François Voruz, Dionysios Neofytos, Christian Van Delden, Johannes Lobrinus, Claudio De Vito, Sonia Macario, Dimitrios Daskalou, Julien W. Hsieh, Minerva Becker, Basile N. Landis

**Affiliations:** 1 Rhinology–Olfactology Unit, Department of Clinical Neurosciences, Clinic of Otorhinolaryngology—Head and Neck Surgery, Geneva University Hospitals, University of Geneva, 1211 Geneva, Switzerland; 2 Department of Internal Medicine, Service of Infectious Diseases, Geneva University Hospitals, University of Geneva, 1211 Geneva, Switzerland; 3 Diagnostic Department, Division of Pathology, Geneva University Hospitals, University of Geneva, 1211 Geneva, Switzerland; 4 Diagnostic Department, Division of Radiology, Geneva University Hospitals, University of Geneva, 1211 Geneva, Switzerland

**Keywords:** invasive fungal rhinosinusitis, mucor, mucormycosis, zygomycosis, sinusitis, immunosuppression, agranulocytosis, black turbinate, MRI

## Abstract

Acute invasive fungal rhinosinusitis (AIFR) is a rare, severe, and life-threatening opportunistic infection associated with high mortality and morbidity. Rapid and accurate diagnosis and treatment are crucial for survival and effective disease management. Diagnosing AIFR is challenging because no single pathognomonic feature exists other than surgical biopsy showing fungal angioinvasion and necrosis. This narrative review focuses on the diagnostic challenges and pitfalls, emphasizing the critical clinical value of magnetic resonance imaging (MRI) for early diagnosis of AIFR. It includes selected cases that illustrate the significance of MRI. When AIFR is suspected, clinical symptoms, nasal endoscopy, blood samples, and facial computed tomography all provide non-specific information. In contrast, MRI can identify signs of devitalized sinonasal mucosa consistent with AIFR. The absence of mucosal enhancement on T1-weighted images, combined with restricted diffusivity, are characteristic MRI features of AIFR. The cases presented underscore the usefulness of MRI in supporting clinical suspicion of AIFR and accurately determining its topography, thereby guiding early surgical biopsies and debridement. In suspected cases of AIFR, MRI serves as a valuable supplementary, non-invasive tool to help determine whether prompt surgical biopsy or debridement is necessary, thereby enhancing early diagnosis and improving survival rates. Therefore, the threshold for conducting an MRI in these cases should be low.

## 1. Introduction

Acute invasive fungal rhinosinusitis (AIFR) is a rare, aggressive, and life-threatening opportunistic infection characterized by the invasion of the sinonasal mucosa by fungal hyphae [1]. This typically unilateral infection occurs solely in immunocompromised patients; adults and children [2]. Those who are most vulnerable include individuals suffering from uncontrolled diabetes mellitus [3], patients with hematological malignancies (e.g., acute myeloid leukemia, myelodysplastic syndrome), and transplant recipients, especially those undergoing allogeneic hematopoietic cell transplantation. In those patients, advanced HIV infection and the use of corticosteroids are common underlying conditions for AIFR [4]. Recently, the COVID-19 pandemic was associated with an increase in the incidence of AIFR in certain countries [5,6,7], although its true incidence remains unknown and is probably underestimated [8]. At our tertiary care hospital, AIFR is primarily encountered in neutropenic patients with hematological malignancies. Even in a tertiary care center with comprehensive access to necessary medical resources, the timely diagnosis remains a challenge, and the estimated survival rate is 50 to 60% [9,10].

Patient survival and effective control rely on prompt diagnosis and timely initiation of appropriate antifungal therapy, along with aggressive surgical debridement. To enhance intravenous access to antifungal medication, a quick surgical approach aims to eliminate necrotic and infectious burdens, thereby reducing the need for mutilating surgery. AIFR lacks pathognomonic symptoms [1], as well as distinctive findings in nasal endoscopy and computed tomography (CT) imaging [11,12]. Symptoms are somewhat nonspecific and include pain, rhinorrhea, swelling, and nerve dysfunctions, which can also occur in acute bacterial infections. Certain blood markers, such as serum galactomannan, may raise suspicion of AIFR if positive but remain nonspecific regarding the exact site of fungal invasion [13]. A multidisciplinary, collaborative, and rapid approach involving specialists from various fields with experience and expertise in managing AIFR facilitates adequate clinical diagnosis and optimized management to minimize morbidity and mortality [8]. In this illustrated narrative review, we examine the diagnostic challenges and pitfalls, emphasizing the critical clinical value of magnetic resonance imaging (MRI) for early diagnosis. We propose a pragmatic algorithm to aid in decision-making for diagnosing AIFR. This article does not address chronic and granulomatous forms of invasive fungal infections [14].

**Figure 1:**
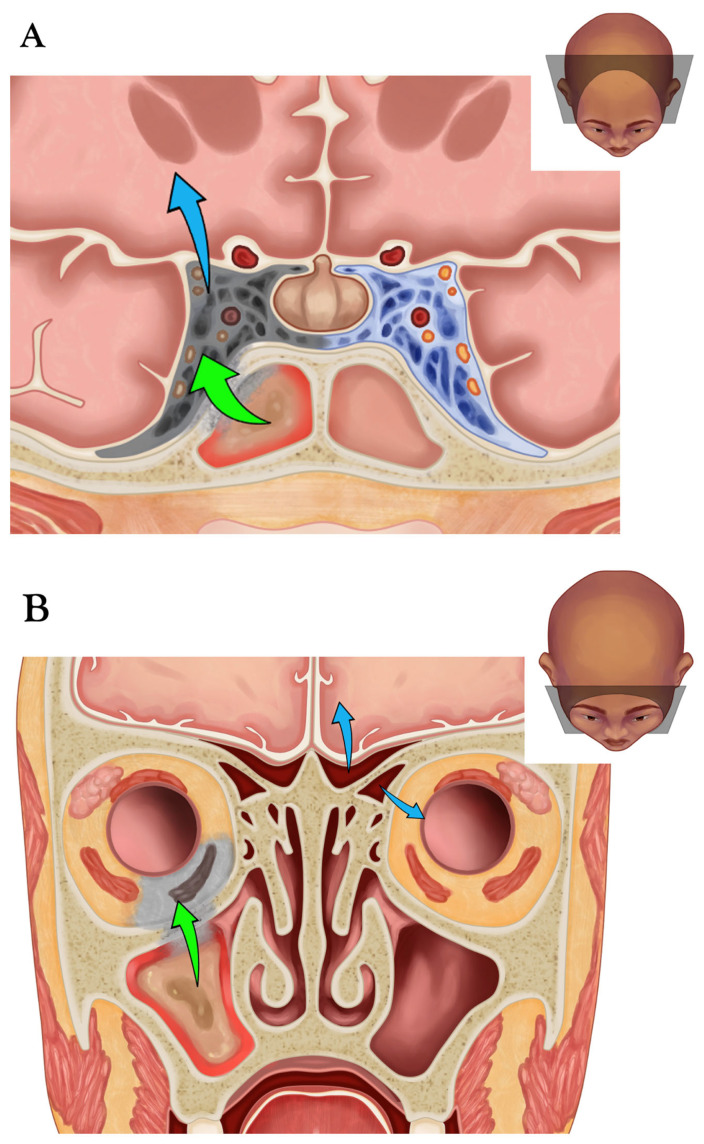

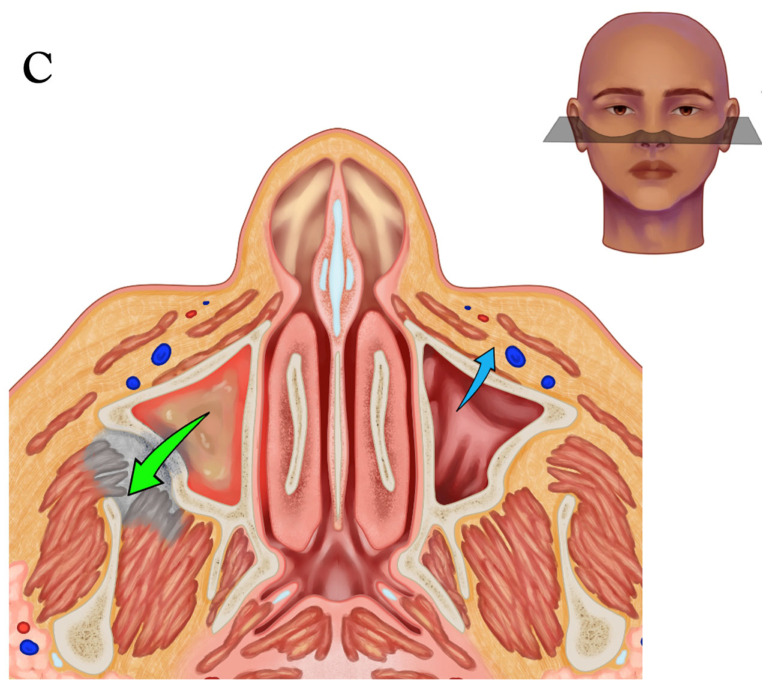
Possible routes for craniofacial invasion by AIFR. (**A**) Coronal section illustrating the invasion route from the right sphenoid sinus through its bony wall to the cavernous sinus (green arrow). The inflamed pink sinus mucosa appears pale gray in the area of necrosis caused by fungal angioinvasion, leading to septic thrombosis and necrosis of the cavernous sinus contents, which results in neuropathies involving cranial nerves III, IV, VI, V_1_, and V_2_. Furthermore, the intracavernous internal carotid artery is often involved. The infection can further spread to the brain (blue arrow). (**B**). Coronal section illustrating the invasion route from the right maxillary sinus to the orbit (green arrow). The inflamed pink sinus mucosa appears pale gray in the area of necrosis caused by fungal angioinvasion, extending through the orbital floor and the periorbita to the extraconal orbital fat and the inferior rectus muscle, resulting in ophthalmoplegia. The infection can further spread to the intraconal fat, reaching the optic nerve sheath and the vasa vasorum of the optic nerve, which can lead to ischemia. Other potential routes include the ethmoid and frontal sinuses to the orbit and the brain (blue arrows). (**C**). Axial section illustrating the invasion route from the right maxillary sinus through its posterolateral wall to Bichat’s fat pad (green arrow), from which the infection can further spread to the pterygopalatine fossa. The inflamed pink sinus mucosa appears pale gray in the area of necrosis due to fungal angioinvasion, extending through the bony wall. Another potential route involves the maxillary sinus leading to the canine fossa (blue arrow).

Several molds, most commonly *Aspergillus* spp. and Mucorales, can be found in the upper respiratory tract of human hosts after airborne spores have been inhaled in the community [1,15,16]. Other species are occasionally reported, such as *Schizophyllum* and *Fusarium* [17]. Nosocomial acquisition of fungal colonization can also occur [18]. In immunocompromised hosts, molds may locally invade the surrounding tissues, leading to AIFR. This is true for all molds, particularly for members of the Mucorales family (*Rhizopus*, *Mucor*, *Rhizomucor*). These pathogens tend to grow rapidly and invade blood vessels, resulting in necrotizing vasculitis, progressive ischemic infarction, and necrosis. In cases of AIFR, the disease can extend along the vessels and through bone foramina to the surrounding tissues in the canine, pterygopalatine, and infratemporal fossae, as well as the intraorbital and intracranial spaces (Figure 1) [19]. Without appropriate treatment, contiguous intraorbital and intracranial spread may occur, leading to cavernous sinus thrombosis and cerebral infarcts, which are responsible for high morbidity and mortality [10].

The treatment of AIFR is threefold: controlling concomitant immunosuppression, antifungal therapy, and debridement [1]. First and foremost, efforts to improve the patient’s immune status should be prioritized, which includes managing diabetes mellitus and optimizing or reducing immunosuppressive treatment, if possible. This is not always feasible, particularly for patients with chemotherapy-related neutropenia, which may persist for several weeks. Additionally, systemic antifungal therapy must be initiated immediately, using, depending on pre-existing prophylaxis and markers suggestive of either *Aspergillus* or Mucorales species, broad-spectrum azoles that cover filamentous fungi, such as isavuconazole or high-dose lipid formulations of amphotericin-B [1,20]. Combination antifungal treatments and local applications [21,22] in association with systemic antifungal treatments have been employed without clear benefit [20,23]. A delay in the initiation of antifungal treatment increases mortality [24,25]. The administration of antifungal treatment is often insufficient since ischemic necrosis due to angioinvasion precludes appropriate antifungal concentration in affected tissue. Therefore, a combined medical and surgical approach is recommended. The latter is an independent predictor of improved survival but is associated with significant morbidity in the craniofacial area, particularly in cases of delayed diagnosis when the anatomical borders of the orbit and skull base have already been crossed [10].

It is important to note that AIFR must not be confused with two non-invasive fungal rhinosinusitis conditions. *Allergic fungal rhinosinusitis* is a benign and potentially destructive eosinophilic primary chronic rhinosinusitis that occurs in immunocompetent patients with hypersensitivity to fungi. The diagnosis relies on histology, and the definitive treatment is surgical, typically accompanied by either local or systemic steroids [1]. *Fungus ball* (also known in the literature as *aspergilloma* or *mycetoma*) is a benign and sometimes asymptomatic secondary chronic rhinosinusitis seen in immunocompetent patients, caused by the accumulation of fungal hyphae and debris within a paranasal sinus (usually the maxillary and sphenoid) without local invasion. The treatment is exclusively surgical, focusing on the removal of the fungus ball [1]. Finally, immunocompromised patients may experience acute bacterial sinusitis with complications that produce symptoms similar to those of AIFR. Complicated bacterial sinusitis may also necessitate surgery, though to a lesser extent and with less radical measures.

## 2. Diagnostic Challenges and Pitfalls

Given the absence of specific clinical symptoms and signs in the early stages, a high level of clinical suspicion is essential for diagnosing AIFR. At the bedside, a key indicator may be significant, deep-seated, persistent sinus pain accompanied by headaches. This type of pain, especially in hematology-oncologic patients with neutropenic fever and CT evidence of sinusitis, should heighten clinical suspicion for AIFR and prompt immediate further investigations. In advanced stages, local mucocutaneous findings, such as necrotic lesions of the nose and paranasal areas, may be observed [20]. This is particularly true for uncontrolled diabetic patients presenting with rhino-orbital mucormycosis. However, not all areas of sinonasal mucosa are easily accessible through nasal endoscopy, particularly the mucus layer within the paranasal sinuses.

**Figure 2:**
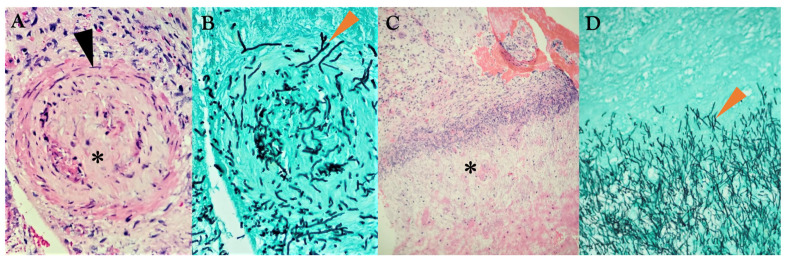
Pathognomonic histological features of AIFR. (**A**). Angioinvasion with *Schizophyllum commune* hyphae within the vessel wall (arrowhead) and lumen (asterisk). H&E staining. Original magnification: 200×. (**B**). The same section highlighted with Grocott staining shows numerous fungal hyphae (arrowhead). Original magnification: 200×. (**C**). Mucosal necrosis (asterisk). H&E staining. Original magnification: 40×. (**D**). Magnification of the same section with Grocott staining, revealing numerous fungal hyphae (arrowhead). Original magnification: 100×.

A multidisciplinary collaborative diagnostic approach is essential, involving specialists from hematology-oncology, infectious disease, otorhinolaryngology, ophthalmology, radiology, and pathology. Each member of this task force should be well-acquainted with the full spectrum of this condition, and evidence-based communication and cooperation are crucial [8]. The definitive diagnosis requires clinical and radiological signs of rhinosinusitis, alongside histopathological necrosis of the mucosa and surrounding tissues with angioinvasion by fungal hyphae (Figure 2) [26]. A nasal swab test for direct microscopy and culture may reveal the presence of filamentous fungal elements, which are highly indicative of AIFR and could be sufficient to initiate systemic antifungal therapy, depending on the clinical context. However, commensal fungi can exist on the nasal mucosal surfaces of healthy individuals, and distinguishing colonization from invasive disease is not feasible. Therefore, a targeted tissue biopsy seeking local fungal invasion is critical for a prompt definitive diagnosis, enhancing the chances of timely intervention and, consequently, survival.

### Several Pitfalls May Slow Down the Diagnosis at Each Step of the Process

**Figure 3:**
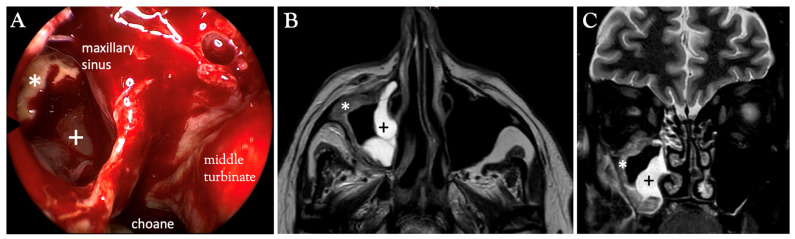
54-year-old male confirmed with right AIFR (*Mucor*). (**A**) Intraoperative endoscopic view after opening the right maxillary sinus. Within it, congested yet still vital sinus mucosa (+) is clearly distinguishable from the whitish necrotic sinus mucosa (asterisk) as indicated by the corresponding MRI ((**B**) axial, (**C**) coronal).

**Clinical evaluation**—At an early stage, the symptoms of AIFR are often minimal and non-specific, including nasal obstruction, painful swelling, clear rhinorrhea, headache, or fever [1,10]. Usually, no purulent discharge is visible during nasal endoscopy in these immunosuppressed patients, particularly in cases of neutropenia. Mucosal necrosis can be noted endoscopically, although devitalized mucosa may also appear pale [27,28] (Figure 3). Deep invasion can also be clinically silent. In more advanced stages, ocular or neurological signs may develop, including double vision, proptosis, ptosis, ophthalmoplegia, facial numbness [1,10], and, rarely, stroke [29,30]. In our experience, pain and swelling in the facial area are the most reliable symptoms suggestive of AIFR at an early stage.

**Laboratory findings**—Serum analysis for the presence of galactomannan can be informative of an underlying invasive fungal infection, predominantly invasive aspergillosis. Galactomannan, detected by an enzyme immunoassay, is a protein produced during hyphal growth, primarily associated with *Aspergillus* spp., but also found in infections caused by other fungal pathogens, such as *Fusarium* spp. or *Alternaria* spp., which may be involved in AIFR. However, members of the Mucorales family do not produce galactomannan, making this test ineffective for diagnosing mucormycosis [1]. Finally, the sensitivity of this assay varies, being higher in patients with neutropenia and significantly lower in other states of immunosuppression, potentially influenced by exposure to azole prophylaxis. Furthermore, a positive test does not help determine the site of infection. Consequently, no biomarker is reliably and specifically available to confirm AIFR [8].

**Surgical biopsy**—Given the large, tortuous surfaces of the sinonasal cavities, blind biopsies are not recommended due to their poor diagnostic yield. Furthermore, these procedures carry a non-negligible risk of morbidity. Therefore, a targeted biopsy of the affected mucosa is essential for a definitive histological diagnosis, necessitating endoscopic sinus surgery under general anesthesia performed by a trained otorhinolaryngologist (Figure 3). However, this procedure may be challenging since thrombocytopenia is often present in hematology-oncologic patients, leading to significant intraoperative and potentially postoperative bleeding, even with the aid of platelet transfusions and fresh frozen plasma. The risk of conducting an invasive diagnostic procedure in thrombocytopenic patients must be carefully weighed against the potential benefits for this high-risk population. Initiating systemic antifungal treatment should not be delayed while awaiting a decision on the necessity of a surgical biopsy. Additional factors, such as clear MRI findings supporting AIFR (see hereafter), can guide the necessity for a surgical procedure. Otorhinolaryngologists with experience and expertise in the management of hematology-oncologic patients can significantly enhance clinical outcomes and survival in these cases. It is also crucial for biopsies to be processed rapidly by an experienced pathologist. The histological identification of filamentous fungal elements showing angioinvasion alongside tissue necrosis caused by microthrombi currently represents the most specific diagnostic indicator [26].

**Figure 4:**
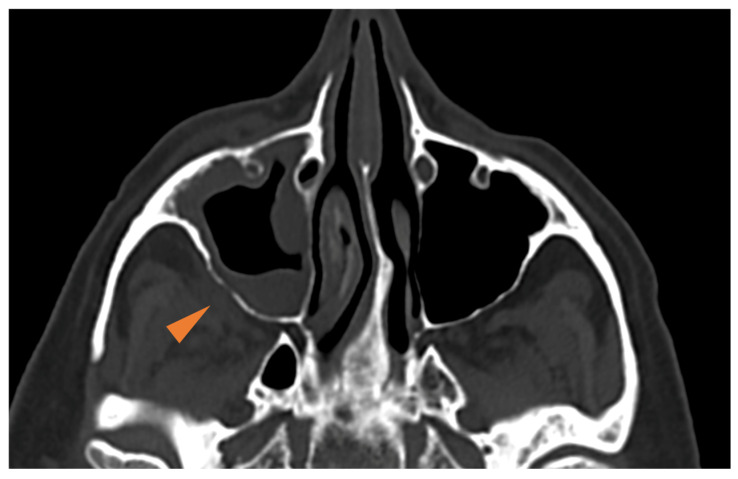
54-year-old male confirmed with right AIFR (*Mucor*). CT axial section showing right periantral fat infiltration (arrowhead) associated with subtle thinning of the posterior maxillary bony wall. See Figure 1C.

**Radiologic findings**—The maxillary sinuses are the most commonly affected, followed by the ethmoid and sphenoid sinuses [31,32]. Unilateral involvement occurs more frequently than bilateral involvement [1]. Facial CT is often conducted as a first-line approach due to its wide availability and rapid execution in emergency situations. In the early stages of AIFR, non-specific mucosal thickening or opacification of the sinuses can be observed, with or without associated air-fluid levels. Subtle bony erosions accompanied by periantral reticulated soft tissue enhancement, and without abscess formation, can be noted in more advanced stages (Figure 4) [3,12,33]. These imaging findings should be considered highly suspicious for AIFR within the appropriate clinical context [3,33,34]. However, facial CT may not reliably differentiate between an acute bacterial infection and AIFR. Clinicians should, therefore, have a low threshold for performing an MRI in patients with abnormalities on CT, as detailed hereafter.

## 3. The Crucial Role of MRI

**Figure 5:**
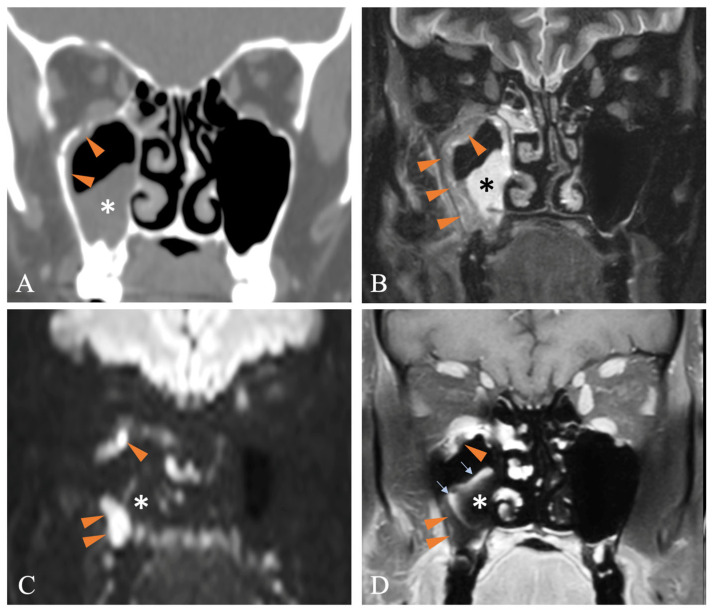
54-year-old male confirmed with right AIFR (*Mucor*). Coronal CT (**A**) and corresponding T2W fat-saturated (**B**), diffusion-weighted (**C**), and contrast-enhanced T1W fat-saturated (**D**) MR images. On CT, there is non-specific opacification of the right maxillary sinus (asterisk) and minimal mucosal thickening along the lateral sinus wall and roof (arrowheads). On MRI, the mucosa covering the roof and lateral sinus wall appears slightly hypointense on the T2W image (arrowheads in (**B**)), shows restricted diffusivity (arrowheads in (**C**)), and the vast majority does not enhance (arrowheads in (**D**)). The non-specific opacification seen in (**A**) corresponds to an inflammatory polyp (large asterisks on (**A**–**D**)) characterized by a very high signal on the T2W image, no restriction of diffusivity, and only thin mucosal superficial enhancement (thin arrows in (**D**)).

**Figure 6:**
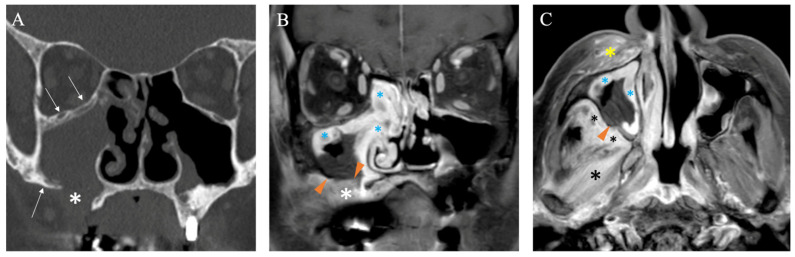
71-year-old female confirmed with right AIFR (*Mucor*). Coronal CT (**A**) shows non-specific opacification of the right maxillary sinus. The bony sinus walls are thickened (arrows), and there is an anterior-inferior defect (white asterisk) caused by a Caldwell-Luc procedure performed in the past. Coronal (**B**) and axial (**C**) contrast-enhanced T1W fat-saturated MR images reveal inflamed, strongly enhancing mucosa (blue asterisks), along with areas of non-enhancing mucosa (arrowheads) that strongly suggest AIFR. There is infectious involvement of the retro-antral fat (small black asterisks), pterygoid muscles (large black asterisk), and subcutaneous cheek fat (yellow asterisk). See Figure 1C.

**Figure 7:**
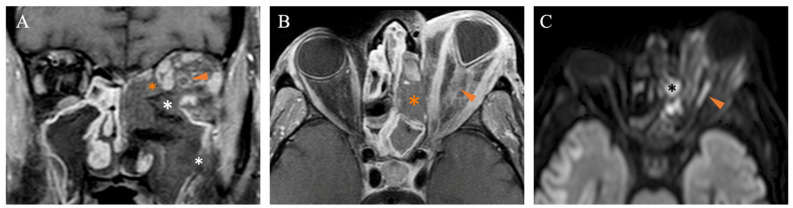
40-year-old male confirmed with left AIFR (*Mucor*). Coronal (**A**) and axial (**B**) T1W fat-saturated images reveal opacification of the maxillary and ethmoidal sinuses. Areas with non-enhancing mucosa are noted in the left maxillary sinus (white asterisks) and the left ethmoid (orange asterisk), strongly suggesting AIFR (See Figure 1B). The infectious involvement of the entire left orbit shows reticulated enhancement. There is also enhancement of the optic nerve sheath (arrowhead) along with significant globe deformation. The axial diffusion-weighted image (**C**) displays restricted diffusivity in the left ethmoid (asterisk) and in the optic nerve (arrowhead), corresponding to mucosal necrosis and optic nerve ischemia, respectively.

**Figure 8:**
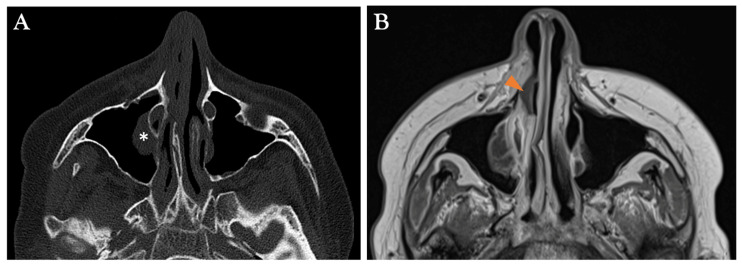
17-year-old male confirmed with right AIFR (*Aspergillus flavus*). (**A**) CT axial section showing the normal anatomical appearance of the nasal fossa, with a small non-specific mucosal thickening in the right maxillary sinus (asterisk). (**B**) The corresponding gadolinium-enhanced T1W MRI axial section reveals a non-enhancing area within the right inferior turbinate mucosa (arrowhead), also referred to as the “black turbinate” sign.

As previously mentioned, nasal endoscopy and CT findings may assist but have limited specific value in diagnosing AIFR. Contrast-enhanced MRI is the preferred imaging modality to support clinical and computed tomography-based suspicion of AIFR and plan surgical biopsies. In addition to being non-irradiating, it provides supplementary topographical information that helps identify necrotic tissue and determine the extent of required surgical debridement [35,36]. On T2-weighted (T2W) sequences, the involved mucosa can exhibit variable signal intensity, whereas contrast-enhanced T1-weighted (T1W) images typically show absent enhancement or a mixed pattern of enhancing and non-enhancing areas in about 80% of cases [32] (Figure 3, Figure 5, Figure 6, Figure 7 and Figure 8). Nevertheless, in approximately 17% of patients with AIFR, the affected mucosa may show strong contrast enhancement, complicating the diagnosis without other MRI signs indicating AIFR [32]. Therefore, identifying non-enhancing sinonasal mucosa on T1W images, with or without deep peri-sinonasal tissue inflammation, plays a key role in diagnosis. Since AIFR is characterized by progressive invasion and tissue necrosis, a sudden and abrupt lack of circumscribed contrast uptake in otherwise homogeneous sinonasal mucosa confirms devitalized areas. This radiological feature is often referred to in the literature as the “black turbinate” sign [37] (Figure 8). However, since AIFR is not confined to the turbinate mucosa and can occur throughout the entire sinonasal tract, this radiological sign is more accurately described as “lack of contrast enhancement” or “absent contrast enhancement” [35]. Although existing data in the literature are based on small patient series, absent contrast enhancement is observed in about 60% of patients with AIFR [32]. Regarding absent contrast enhancement in the turbinates, interpreting it as a sign of devitalized tissue should be approached with caution, as this feature can also be present in up to 30% of patients without AIFR [38]. Radiological features that differentiate the physiological non-enhancing turbinate from the non-enhancing turbinate indicative of AIFR include preserved peripheral enhancement and thin septations, though these features can sometimes be quite subtle [38]. Another characteristic feature of AIFR is restricted diffusivity, which is seen in about 90% of cases on diffusion-weighted imaging (DWI). While restricted diffusivity can also occur in non-invasive fungal diseases and sinusitis with pus accumulation, the combination of absent mucosal enhancement on T1W images and restricted diffusivity enables accurate diagnosis of AIFR (Figure 5 and Figure 7).

The involvement of retroantral fat is an important diagnostic feature that, as mentioned above, can also be observed on CT in more advanced cases of AIFR [3,33] (Figure 4). On T1W images, there is diffuse and pronounced contrast enhancement of the retroantral fat in about 30% of patients with AIFR (Figure 6C), while enhancement in the pterygopalatine fossa occurs in approximately 7% of cases [32]. Since the pterygopalatine fossa serves as a natural conduit for the spread of infection intracranially (Figure 1C), its involvement is concerning [39]. It is important to note that contrast enhancement of the retroantral fat and pterygopalatine fossa is best assessed using fat-saturated T1W sequences.

Orbital (Figure 7) and intracranial (Figure 1A,B) involvement tend to occur later in the disease process. However, involvement of the extraconal and intraconal fat, extraocular muscles, or optic nerve is seen in up to 83% of patients with AIFR at initial MRI, and involvement of extradural/intracerebral and cavernous sinus invasion in 5 to 10% of patients, respectively [32]. Orbital involvement is most often seen as a diffusely enhancing inflammatory process in T1W images, and dural thickening and enhancement are common accompanying features. At MRI, cavernous sinus invasion is seen as a filling defect on contrast-enhanced T1W images and angiography sequences. Angioinvasion and fungal vasculitis typically present as thrombosis, stenosis/occlusion of the internal carotid artery and other intracranial arteries. The consequence of angioinvasion of the central artery of the retina or the vasa vasorum of the optic nerve is ischemia of the nerve itself, which manifests with restriction of diffusivity on DWI sequences [40] (Figure 7).

**Figure 9:**
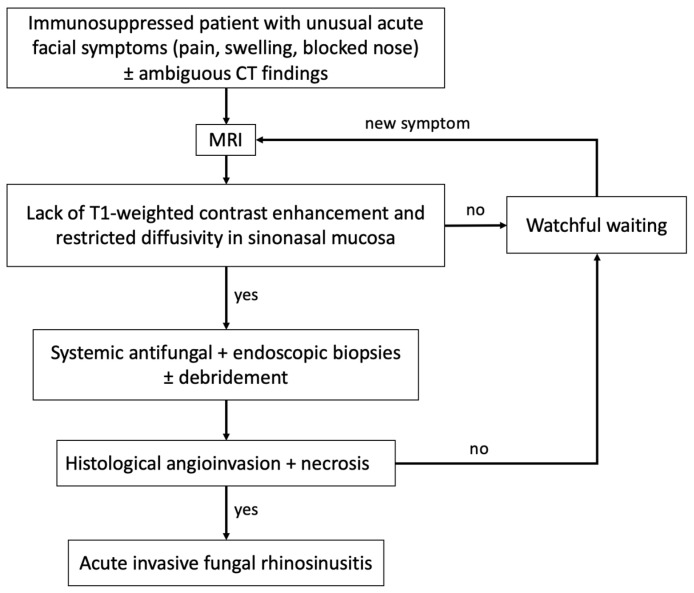
Decision-making algorithm for diagnosing AIFR.

Non-enhancing sinonasal mucosa on MRI in a symptomatic immunosuppressed patient strongly suggests underlying tissue ischemia due to fungal invasion. This sometimes subtle sign of mucosal devitalization should prompt a multidisciplinary decision to perform endoscopic biopsies and debridement of diseased tissue, which may still be confined to the lumen of the sinonasal cavities. Examples of MRI features that have led to the definitive diagnosis of AIFR are shown in Figure 5, Figure 6, and Figure 8, contrasting with their corresponding non-specific CT presentation. However, in addition to sometimes providing hints for underlying AIFR in the early investigation process, the CT provides a superior understanding of bone architecture and surgical landmarks, making it complementary for planning endoscopic sinus surgery [12]. In summary, MRI plays a crucial role in the early individualized diagnostic process of AIFR, facilitating timely planning of efficient surgical biopsies for a prompt and definitive diagnosis. A pragmatic decision-making algorithm is proposed in Figure 9.

## 4. Future Directions

Advancements in diagnosing AIFR with MRI increasingly emphasize integrating artificial intelligence (AI) and radiomics to enhance diagnostic capabilities. AI-driven algorithms, designed to identify subtle early-stage imaging patterns, hold the potential to boost diagnostic accuracy and facilitate earlier therapeutic intervention significantly. Radiomic analysis, leveraging high-dimensional data extracted from imaging, could further refine the characterization of disease-specific features. Progress in MRI technology, combined with AI, aims to make precise imaging more accessible, even in settings with limited resources. Standardized imaging protocols and robust radiomic frameworks will be key to harmonizing diagnostics across institutions. Large-scale, multicenter prospective studies are essential to validate these technologies and seamlessly integrate them into clinical workflows, ultimately improving patient outcomes.

## 5. Conclusions

AIFR requires timely diagnosis to reduce its high morbidity and mortality rates. Medical experience and interdisciplinary expertise across all specialties involved in patient care are paramount for optimized clinical outcomes. The clinical suspicion of AIFR in symptomatic immunocompromised patients should be high, and the most reliable early symptoms include pain and swelling in the facial region. MRI is crucial for identifying early signs of devitalized sinonasal mucosa, enabling targeted surgical biopsies and prompt diagnosis; therefore, the threshold for performing an MRI in suspected AIFR cases should be low.

## Data Availability

No new data were created or analyzed in this study. Data sharing is not applicable to this article.
